# Supplementary material for: Hydrogels from the Assembly of SAA/Elastin-Inspired Peptides Reveal Non-Canonical Nanotopologies
Source: Molecules. 2022 Nov 15;27(22):7901. doi: 10.3390/molecules27227901 (PMC9698559; doi:10.3390/molecules27227901)
Supplement: Supplementary file 1 [file molecules-27-07901-s001.zip › molecules-1978824-supplementary.pdf]

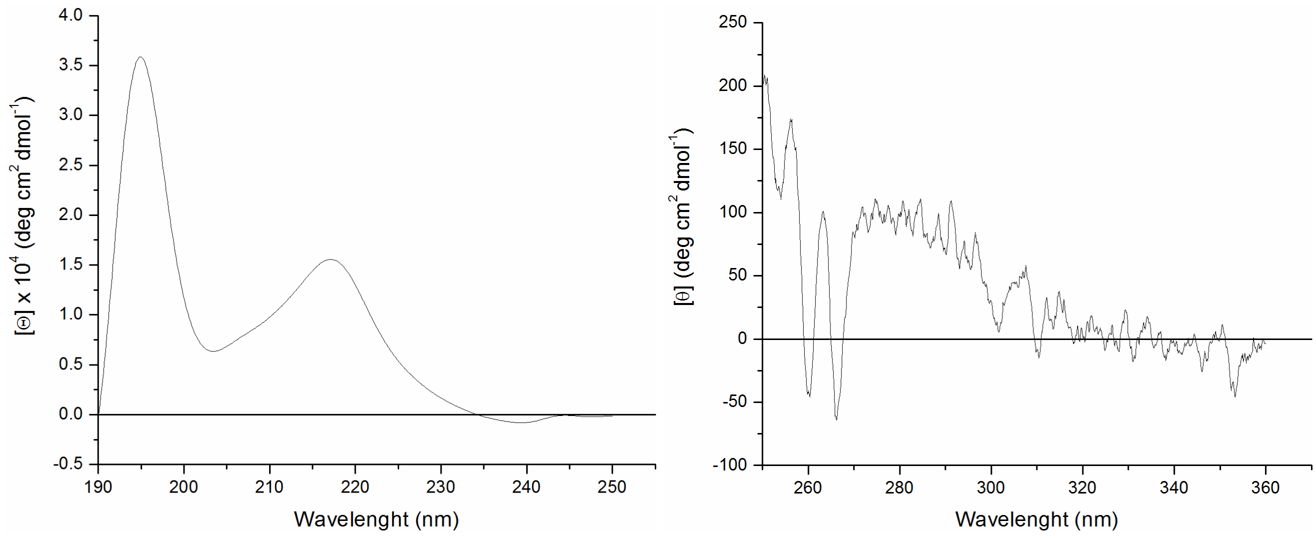

(**a**) (**b**)

Figure S1: Far UV (a) and Near UV (b) CD spectra of P5 peptide recorded respectively at 0.01% w/v and 0.1% w/v.

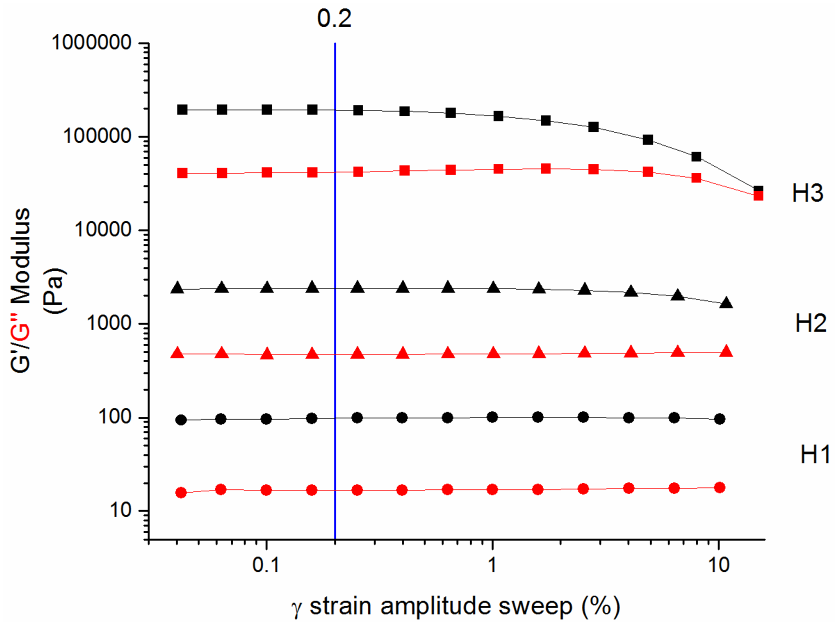

Figure S2: Strain amplitude sweep (γ = 0.04–14%) at constant angular frequency (ω = 1 rad s^{-1}) on H1, H2 and H3 hydrogels are shown to identify the linear viscoelastic region (LVR).

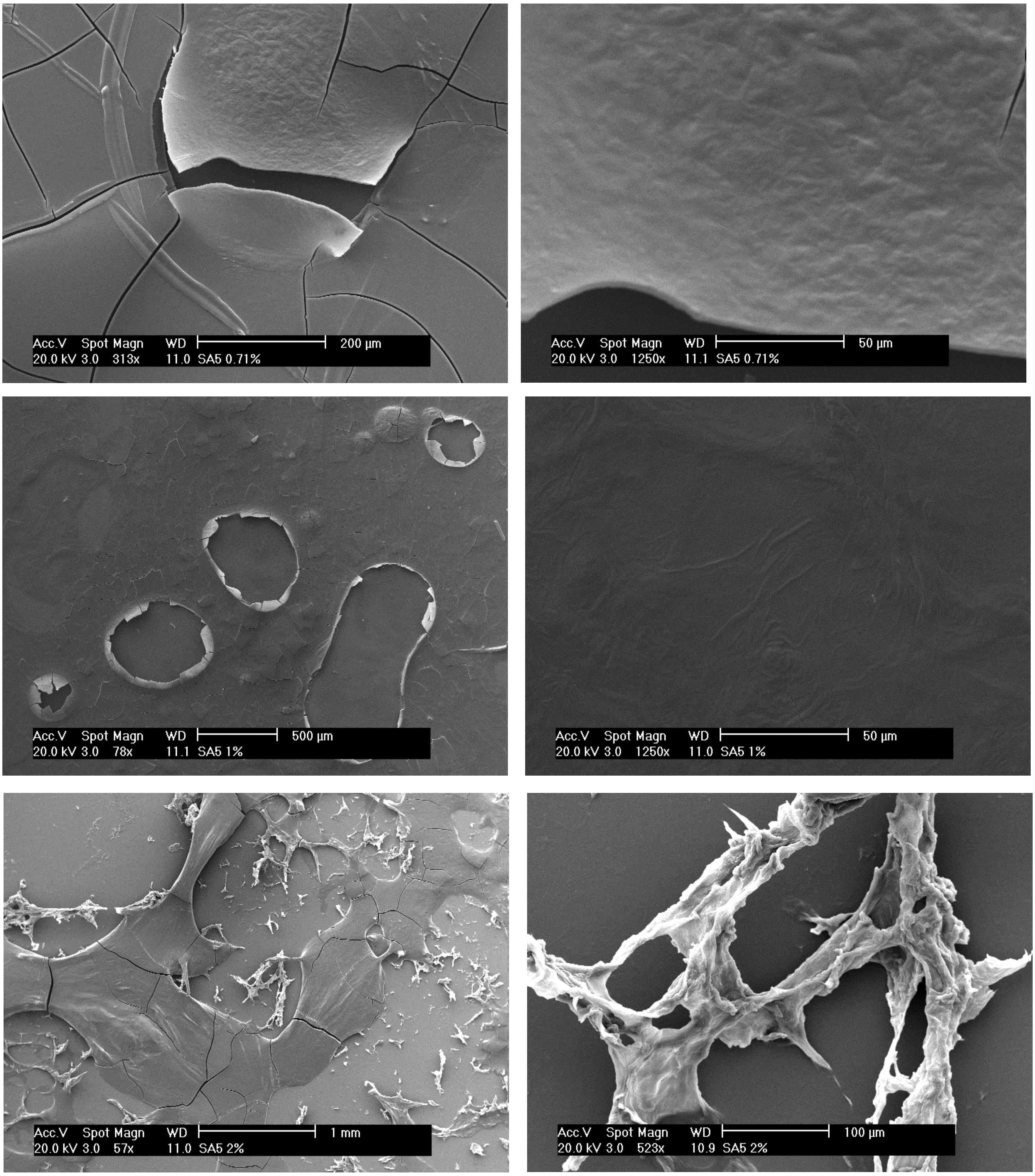

Figure S3: SEM images of H1 (first row) H2 (second row) and H3 (third row) hydrogels acquired after freeze drying of the hydrogels on the silicon substrate.

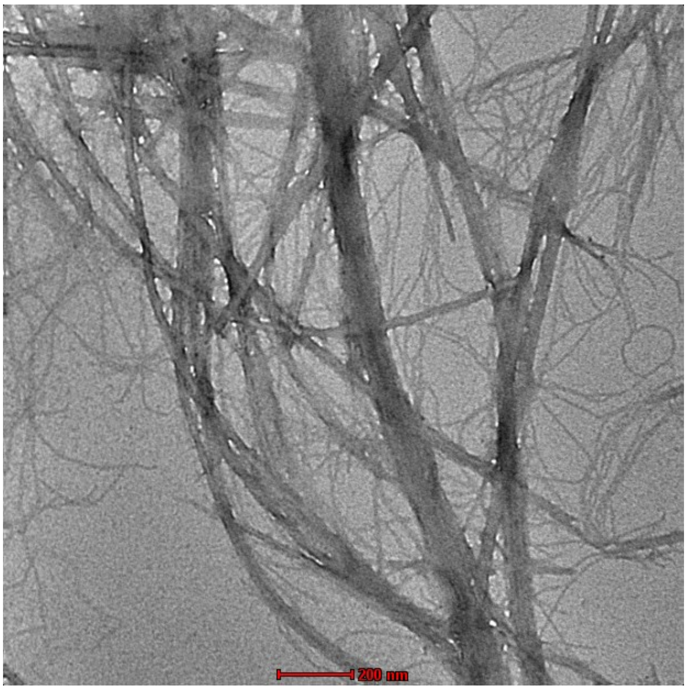

Figure S4: TEM image of H3 hydrogel (2 wt% P5).

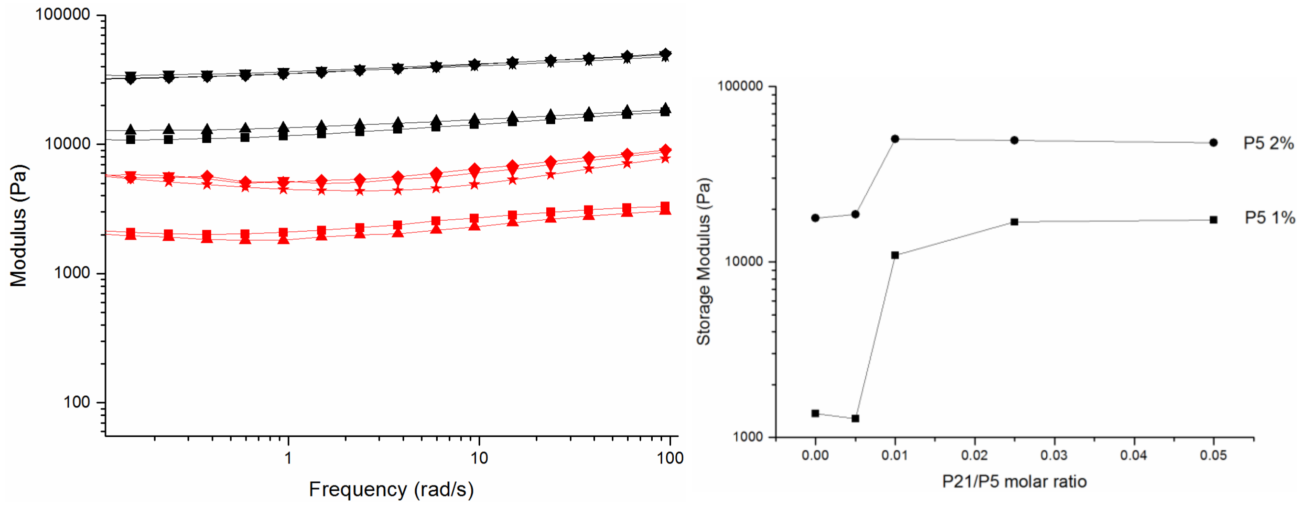

(a) (b)

Figure S5: Rheology of hydrogels of P5 2% (w/v) with different concentration of P21. (a) Dynamic frequency sweep at 0.2% of strain of H3(■), H8 (▲),H9 (♦), H10 (▼)and H11 (★) hydrogels. Black symbols report storage moduli (G'), while red symbols report loss moduli (G''); (b) Storage modulus (G') of hydrogel at 1% (•) and 2% (■) of P5 as a function of concentration of P21 peptide recorded at 0.2 % strain and 9.4 rad s^{-1}.

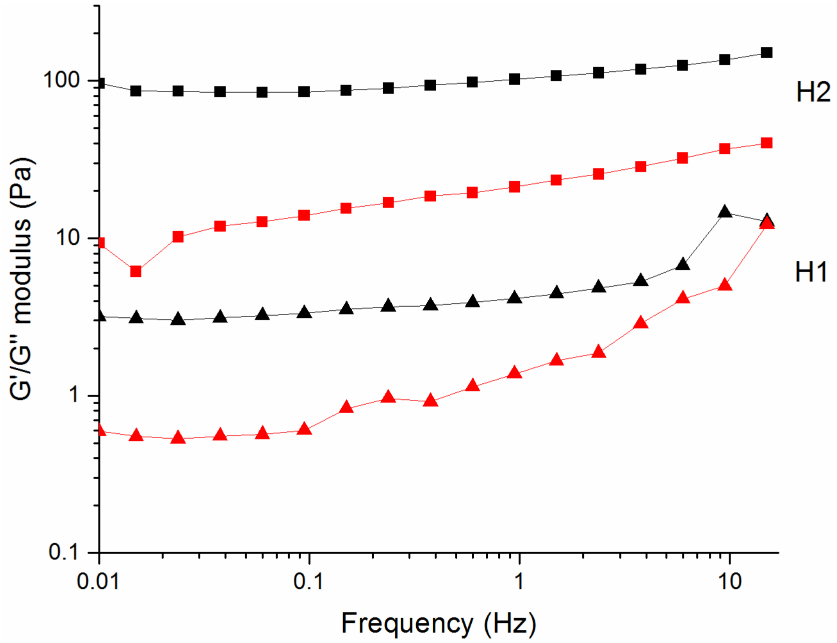

Figure S6: Dynamic frequency sweep at 0.2% of strain of H1(▲), H2 (■) hydrogels formed in 10% AcOH. Black symbols report storage moduli (G′), while red symbols report loss moduli (G′′).

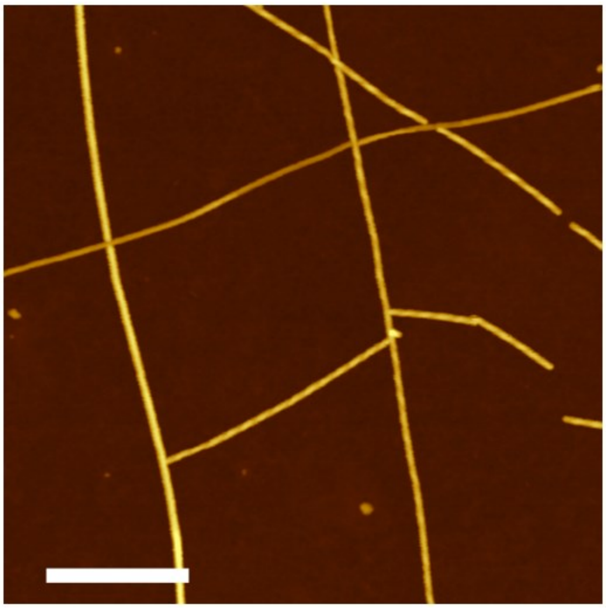

Figure S7: AFM image of hydrogel formed by 1 wt% P5 in 10% (m/v) AcOH . The bar represents 500 nm.

a)

b)

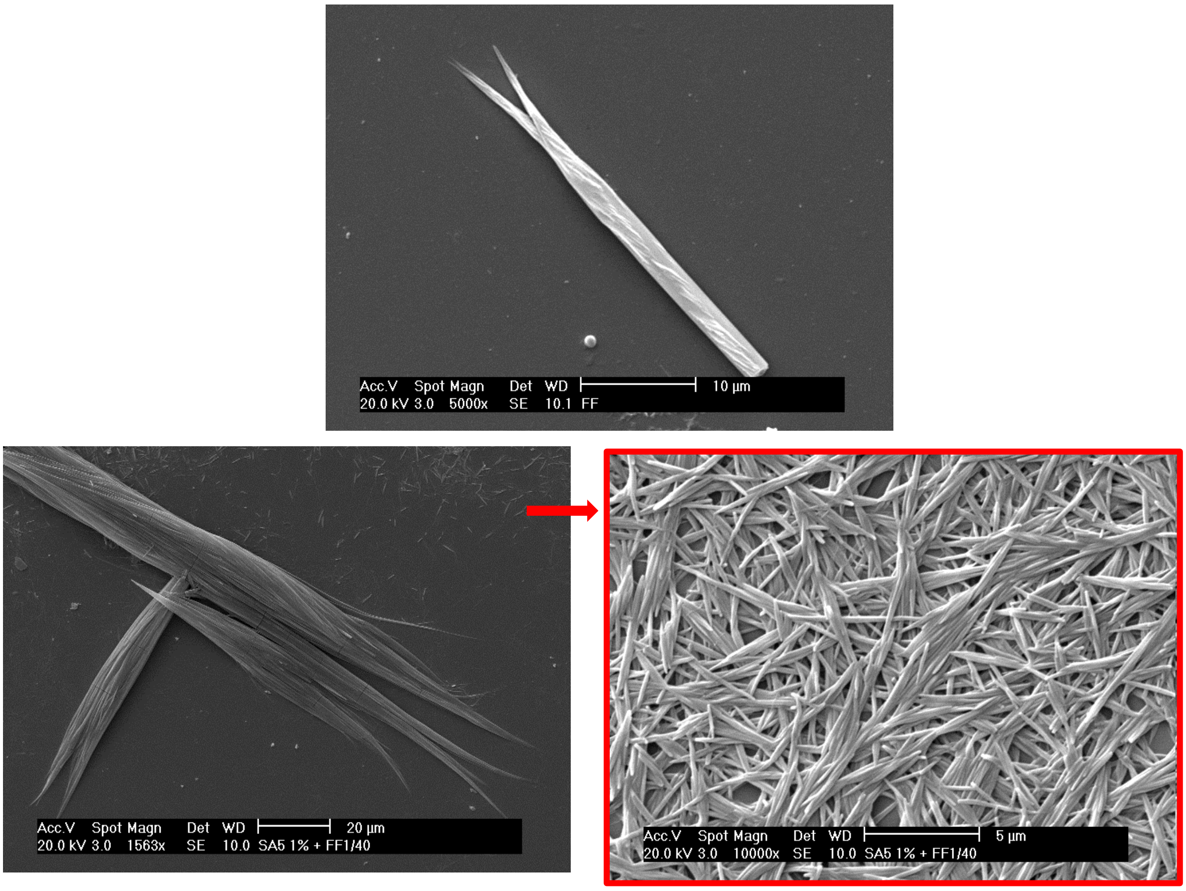

Figure S8: SEM images of a) FF nanotubes formed by following the protocol of Song et al. [34]; b) HF4 hydrogel formed by P5 at 1% (m/v) and FF at 0.25% (m/v); on the right the region indicated by the red arrow at higher magnification.

a)

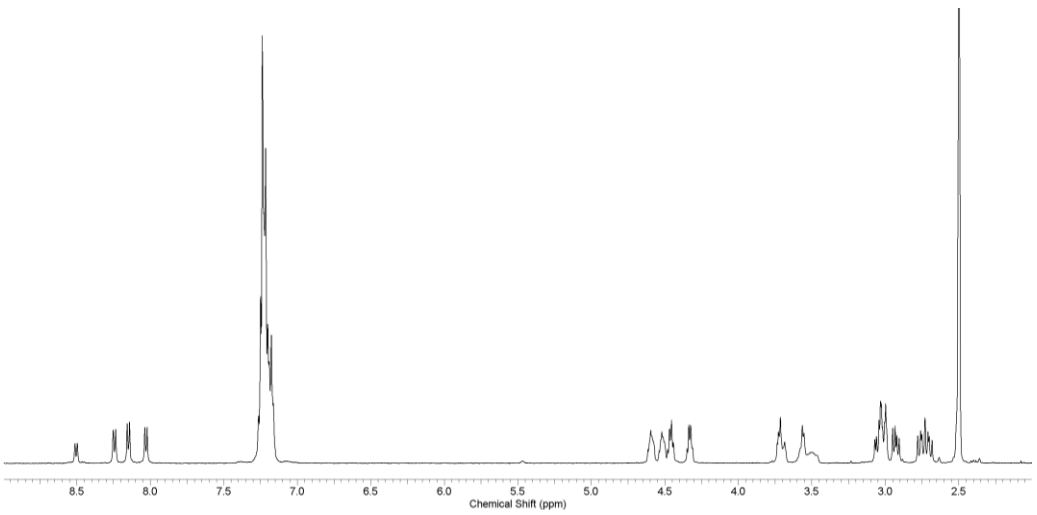

b)

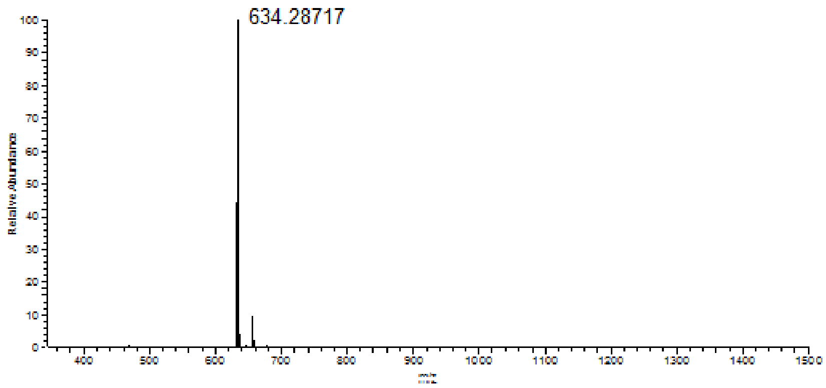

Figure S9: a) ^{1}H-NMR spectrum of P5 recorded in dmso-d_6 at 298K; b) HRESI-MS spectrum of P5.

a)

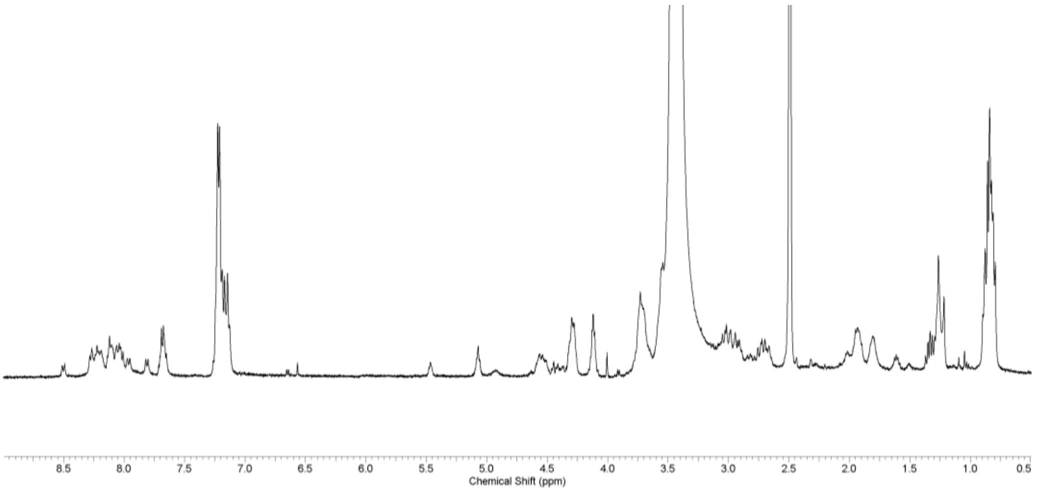

b)

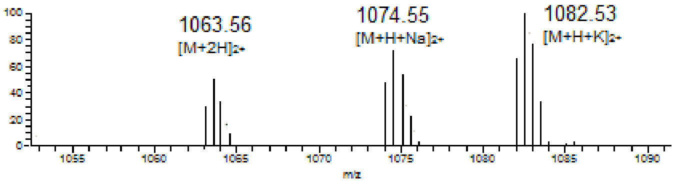

Figure S10: a) ^{1}H-NMR spectrum of P21 recorded in dmso-d_6 at 298K; b) ESI-MS spectrum of P21.

a)

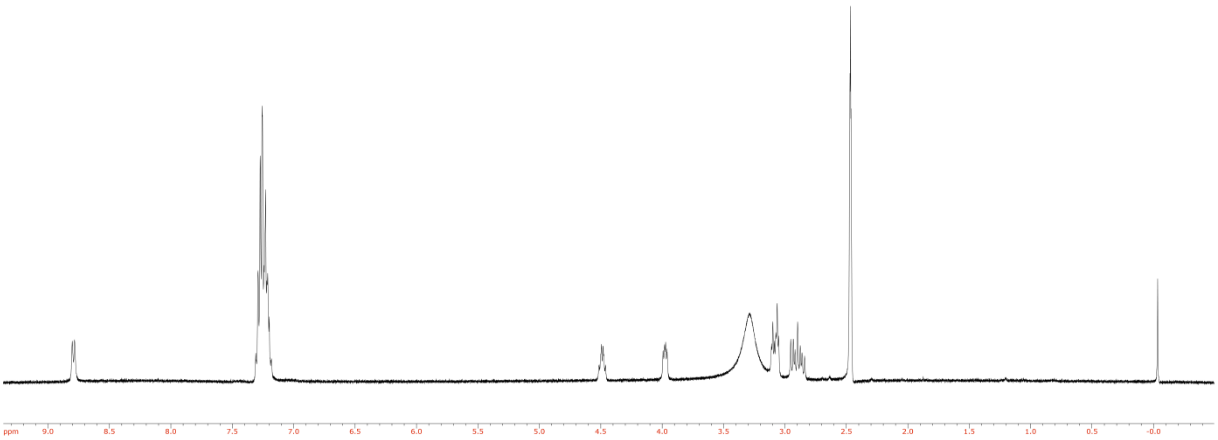

b)

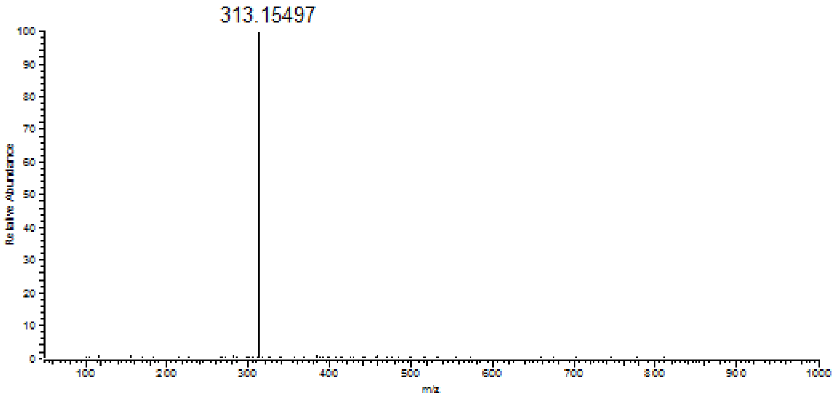

Figure S11: a) ^{1}H-NMR spectrum of FF recorded in dmso-d_6 at 298K; b) ESI-MS spectrum of FF.
